# PUResNetV2.0: a deep learning model leveraging sparse representation for improved ligand binding site prediction

**DOI:** 10.1186/s13321-024-00865-6

**Published:** 2024-06-07

**Authors:** Kandel Jeevan, Shrestha Palistha, Hilal Tayara, Kil T. Chong

**Affiliations:** 1 Graduate School of Integrated Energy-AI, Jeonbuk National University, Jeonju, 54896 South Korea; 2 Department of Electronics and Information Engineering, Jeonbuk National University, Jeonju, 54896 South Korea; 3 School of International Engineering and Science, Jeonbuk National University, Jeonju, 54896 South Korea; 4 Advanced Electronics and Information Research Center, Jeonbuk National University, Jeonju, 54896 South Korea

## Abstract

Accurate ligand binding site prediction (LBSP) within proteins is essential for drug discovery. We developed ProteinUNetResNetV2.0 (PUResNetV2.0), leveraging sparse representation of protein structures to improve LBSP accuracy. Our training dataset included protein complexes from 4729 protein families. Evaluations on benchmark datasets showed that PUResNetV2.0 achieved an 85.4% Distance Center Atom (DCA) success rate and a 74.7% F1 Score on the Holo801 dataset, outperforming existing methods. However, its performance in specific cases, such as RNA, DNA, peptide-like ligand, and ion binding site prediction, was limited due to constraints in our training data. Our findings underscore the potential of sparse representation in LBSP, especially for oligomeric structures, suggesting PUResNetV2.0 as a promising tool for computational drug discovery.

**Supplementary Information:**

The online version contains supplementary material available at 10.1186/s13321-024-00865-6.

## Introduction

Proteins are dynamic molecules that play essential roles in various biological processes by interacting with other molecules, such as organic compounds, nucleotides, metal ions, and other proteins. A full understanding of the function of a protein often requires the identification of its ligand binding sites, which are specific sites on a protein that interact with ligand molecules. A classic example of the importance of understanding protein‒ligand binding sites is the development of targeted therapies in the field of oncology. Precise knowledge of binding sites [1, 2] has allowed for the creation of drugs that specifically target and inhibit cancer-promoting proteins, revolutionizing cancer treatment. Furthermore, insights into the binding sites of enzymes involved in bacterial replication have facilitated the development of potent antibiotics. These examples underline the critical role of accurate protein‒ligand binding site identification in scientific and therapeutic advancements. However, the experimental determination of binding sites, such as by mass spectrometry and mutagenesis, is costly and time-consuming, necessitating the development of computational methods for ligand binding site prediction (LBSP).

Over the years, a plethora of computational methodologies have emerged to improve LBSP, including geometry-based, energy-based, consensus-based, and template-based paradigms. While these paradigms have advanced the field of LBSP, they come with their own sets of limitations. For instance, spatial geometry-based methods [3–9] rely heavily on intricate geometric calculations derived from protein structure information, which are computationally expensive and may not always accurately capture the dynamic nature of protein‒ligand interactions. Energy-based techniques [10–12] involve detailed calculations of the interaction energies between proteins and chemical probes, but these methods can struggle with multisite interactions and may not adequately represent all biological conditions that influence these interactions. Template-based methods, whether they are evolutionary-based methods [13] sequence-based approaches [14] or structure-based techniques [15, 16] are heavily dependent on the quality and availability of reference datasets and may overlook novel binding sites that do not match known templates. These challenges highlight the need for the development of more advanced, efficient methods, such as those based on machine learning and deep learning, for LBSP.

The ever-increasing volume of data in the LBSP field has enabled significant advances through the incorporation of machine learning and deep learning techniques. Notable machine learning methods [17, 18] critically hinge on the accuracy of the designed features and can often lead to false-positive predictions, such as the identification of regions that are not feasible targets for drug interactions. Deep learning methods [19–23] that do not necessitate manual feature engineering, employ 3D convolutional neural networks (CNNs) that represent protein structures as fixed-sized voxels. In general, these methods can be broadly categorized into two distinct groups based on their approach to problem formulation: binding pocket prediction and binding residue prediction.

In the case of binding pocket prediction, the focus is to identify potential pockets on the protein structure where ligands could bind. P2rank and DeepSurf calculate the Solvent Accessible Surface (SAS) points and predict the ligandability score of these points. P2Rank employs Random Forest Classifiers, while DeepSurf uses 3D CNN for this purpose. Both methods then cluster SAS points based on ligandability scores to form and rank predicted pockets. In other hand, DeepSite and PUResNetV1.0 conceptualize protein structures as 3D images, where each voxel represents atoms. DeepSite adopts a subgrid sampling strategy using a sliding window with a step of four voxels and employs deep convolutional neural networks (DCNN) to classify these subgrids as being proximal to the actual binding pocket whereas PUResNetV1.0 utilizes a 3D Segmentation technique based on the UNet architecture, classifying each voxel to determine whether it belongs to the binding pocket.

In contrast, binding residue prediction methods such as DeepCSeqSite and GRaSP specialize in identifying specific residues on the protein surface that are likely to engage in ligand binding. DeepCSeqSite embeds each residue in a multidimensional feature space, comprising seven types of features. Utilizing a 1D DCNN, DeepCSeqSite classifies each residue as either a binding or non-binding residue, effectively discerning the potential interaction sites on the protein surface. In other hand, GRaSP adopts a comprehensive approach by generating a feature vector for each residue, employing the Extremely Randomized Trees algorithm, GRaSP predicts the likelihood of each residue being involved in ligand binding. These diverse methodologies, from P2Rank and DeepSurf’s solvent accessible surface analysis to DeepCSeqSite and GRaSP’s intricate residue-level feature engineering, collectively represent significant strides in LBSP. They demonstrate how leveraging large datasets and complex structural features through advanced computational techniques can overcome the limitations of traditional methods, leading to more accurate and insightful predictions in protein–ligand interaction studies.

Despite these advancements, deep learning techniques are significantly impeded by the sparse nature of protein structures. Here, ‘sparse nature’ refers to the fact that protein structures are mostly empty space, with atoms occupying only a small fraction of the total volume. Typically, these techniques utilize dense representations of protein structures as fixed-sized voxels, much like the pixels in a 3D image. However, this approach has two main drawbacks. First, it involves substantial computational costs, as it requires information to be stored and processed for all voxels, including those that do not contain any atoms. Second, it can lead to a loss of information because proteins have diverse, complex shapes that cannot be accurately represented within fixed-size voxels. Thus, dense representations are less suited for modeling the full complexity of protein structures given their inherent sparsity.

Applying sparse representation techniques to protein structures finds parallels in fields where high-dimensional data are represented in a sparse manner to perform more effective computations. Notably, light detection and ranging (LiDAR)-based semantic segmentation [24, 25] in autonomous vehicle navigation and robotics is a pertinent example. LiDAR semantic segmentation labels each point in a sparse 3D point cloud generated from LiDAR sensors with a class label that describes the object to which it belongs (such as a road, a pedestrian, a vehicle, etc.) The challenge lies in the sparsity of the given point cloud data, like the sparse nature of protein structures. In the realm of LBSP, one can draw an analogy where atoms in a protein structure are equivalent to points in a LiDAR point cloud, and the goal is to classify which of these atoms belong to the binding site; this Minkowski Convolutional Neural Network (MCNN), a specific type of sparse convolutional neural network that operates on a Minkowski SparseTensor, is particularly suitable for handling such tasks.

In this work, we introduce ProteinUNetResNetV2.0 (PUResNetV2.0), a cutting-edge LBSP approach that fundamentally addresses the inherent sparsity of protein structures, which is a major obstacle in the field. Inspired by LiDAR semantic segmentation, our strategy is centered around representing protein complexes as Minkowski SparseTensors and utilizing MCNNs. The developmental workflow encompasses five stages: generating training data by applying a tailored parser for Minkowski SparseTensor representations of the protein structures obtained from the RCSB [26] database based on information provided in BioLip [27] database; implementing PUResNetV2.0 based on MCNNs; optimizing PUResNetV2.0 using Optuna [28]; evaluating PUResNetV2.0 in terms of success rate based on the distance from the center of the predicted binding pocket to the center of the ligand (DCC) and the minimum distance from the center of the predicted binding pocket to any atom in the ligand (DCA), precision, recall, F1 score, and MCC; and deploying PUResNetV2.0 accessible at https://nsclbio.jbnu.ac.kr/tools/jmol. We show that by representing protein structures as Minkowski SparseTensors, PUResNetV2.0 exhibits remarkable capabilities in terms of handling diverse scenarios, such as oligomeric structures and structures interacting with peptides. Furthermore, PUResNetV2.0 outperformed established methods such as P2Rank, DeepSurf, PUResNetV1.0, DeepSite and GRaSP, as evidenced by evaluations across four distinct benchmark datasets: Coach100, which focuses on monomeric protein structures; Holo801, featuring ligand-bound oligomeric structures; Apoholo45, encompassing both ‘apo’ and ‘holo’ protein structures; PDBBind1681, aims at providing high-quality protein complexes, making it a promising tool in the realm of LBSP.

## Materials and methods

### Data acquisition and processing

**Fig. 1:**
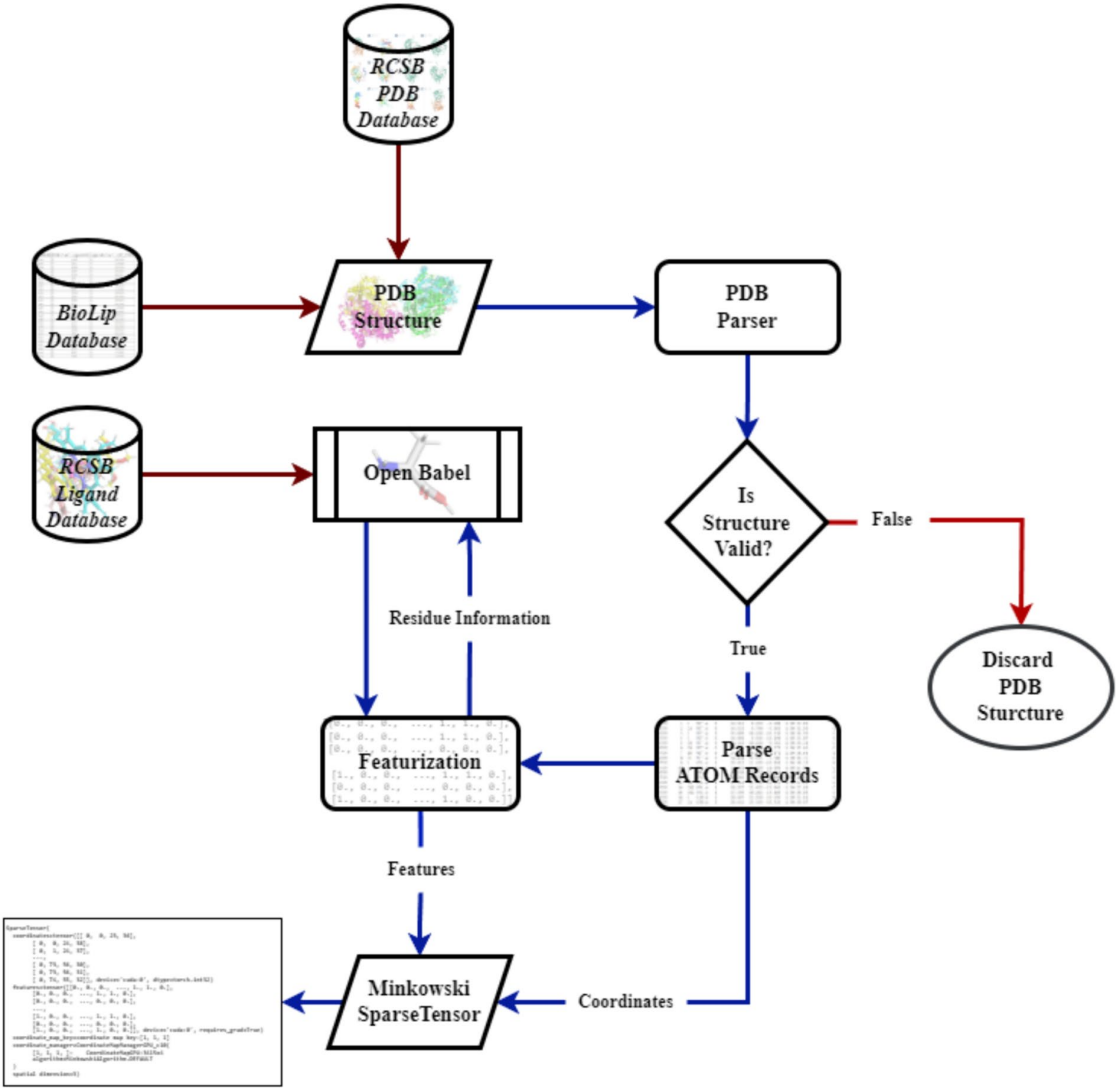
Flowchart illustrating the overall process of preparing the training dataset. We initiate the process by procuring protein structures from the esteemed RCSB PDB database using the BioLip database as a reference. Subsequently, these structures undergo a parsing process using our customized PDB parser, followed by featurization through Open Babel. The final step involves the transformation of these structures into Minkowski SparseTensor representations. Within the figure, brown arrows signify the acquisition of information from external databases, blue arrows illustrate the directional flow of data processing, and red arrows denote the endpoints of data flows.

In this study, as shown in Fig. 1, we acquired a nonredundant set of biologically relevant protein–ligand interactions information from the BioLip database. We then downloaded the relevant protein structures from the RCSB database. We discarded any structures that had resolutions above 2 Å, that contained multiple models with different numbers of atoms, or that included DNA or RNA. Next, we parsed the atomic records according to the specifications mentioned in the WorldWide Protein Data Bank (wwPDB) [29]. Each parsed atom was featurized using Open Babel [30, 31]; this process entailed downloading the residues (in SDF format) from the RCSB ligand database (https://www.rcsb.org/ligand/) and loading them as Open Babel molecule objects. This process allowed us to acquire a diverse and accurate dataset for the experiment.

To construct a sparse representation model, we represented each protein structure as a Minkowski SparseTensor using atomic coordinates and the associated features and labels to formulate a semantic segmentation problem. The featurization process was carried out in the same way as that used by our previous method (PUResNetV1.0), in which each atom was described based on nine atomic features, namely, hybridization, heavy atoms, heteroatoms, hydrophobia, aromatics, partial charges, acceptors, donors, and rings. Consequently, we represented each protein structure as a sparse tensor with a matrix C and a matrix F.

$$C = \left[ {\begin{array}{*{20}c} {x_{1} } \\ \vdots \\ {x_{N} } \\ \end{array} \begin{array}{*{20}c} {y_{1} } \\ \vdots \\ {y_{N} } \\ \end{array} \begin{array}{*{20}c} {z_{1} } \\ \vdots \\ {z_{N} } \\ \end{array} \begin{array}{*{20}c} {t_{1} } \\ \vdots \\ {t_{N} } \\ \end{array} \begin{array}{*{20}c} {b_{1} } \\ \vdots \\ {b_{N} } \\ \end{array} } \right]\quad {\text{and}}\; F = \left[ {\begin{array}{*{20}c} {f_{1}^{T} } \\ \vdots \\ {f_{N}^{T} } \\ \end{array} } \right],$$

where (*x*_*i,*_* y*_*i*_*, *_*Zi*_) denotes the 3D coordinates of the ith atom of the t_i_th protein structure in the b_i_th batch and f_i_ is the feature vector of the ith atom.

For each protein structure, if any atom was within 5 Å of the ligand atom, then it was labeled as a binding site atom; the binding site atoms were represented as a matrix L.

$$L = \left[ {\begin{array}{*{20}c} {l_{1}^{1} } \\ \vdots \\ {l_{N}^{T} } \\ \end{array} } \right]\quad {\text{where}}\;l_{i} = \left\{ {\begin{array}{*{20}ll} {1,} & {if\;the\;i{\text{th}}\;atom\;is\;a\;binding\;atom} \\ {0,} & {otherwise} \\ \end{array} } \right\}.$$

Finally, we prepared a dataset of 61,691 protein complexes, which included 25,780 biologically relevant small molecule binding sites. Careful curation was performed to exclude HETATOM record from the PDB file. Overall, the protein complexes in this dataset were sourced from 4729 different protein families, providing a diverse set of protein structure for the experiment.

### Curating the benchmark datasets

To conduct a comprehensive evaluation of diverse methodologies, we generated three benchmark datasets, Holo801, Coach100 and PDBBind1681, which were derived from the extensively employed Holo4k [32], Coach420 and PDBBind [33] datasets, respectively. To facilitate an accurate comparison among various methods, we eliminated the protein structures found in both our training datasets and the sc-PDB [34] dataset. Consequently, the newly formed Holo801, Coach100 and PDBBind1681 datasets comprised 801, 100 and 1681 protein complexes, respectively.

Furthermore, we established the Apoholo45 dataset derived from D3PM [35], an extensive collection encompassing 45 pairs of bounded and unbounded structures. This dataset was curated excluding any protein structures or structures with binding sites that were present in the PUResNetV2.0 training dataset.

### Model

**Fig. 2:**
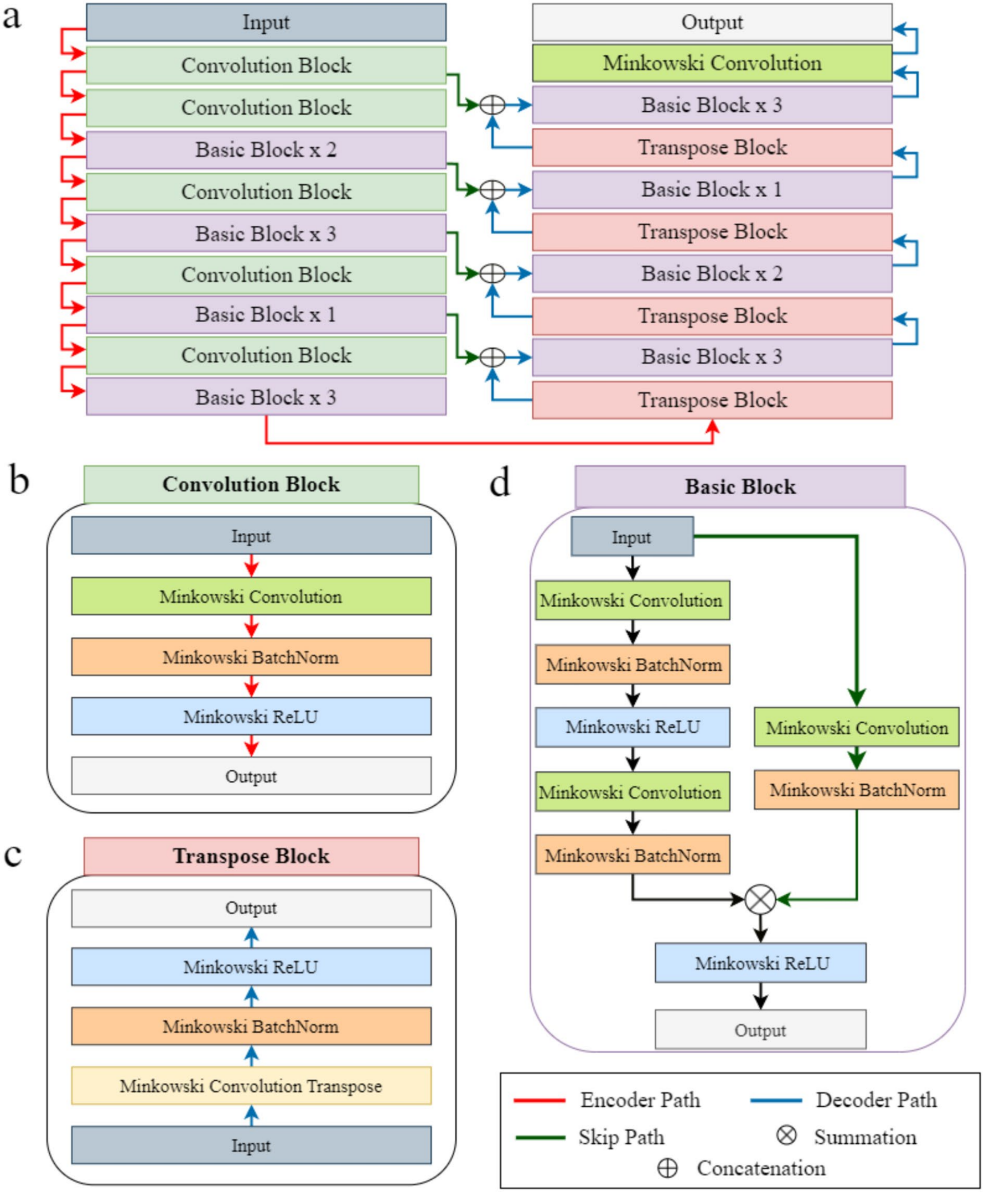
Key components of the PUResNetV2.0 architecture. **a** The overall architecture, highlighting the integration of an encoder path for input downsampling and a decoder path for upsampling the feature maps, with skip connections between the corresponding blocks in both paths. **b** Illustration of the convolution block used within the encoder for input downsampling and feature extraction, which is composed of Minkowski convolutional layer, batch normalization layer, and ReLU activation function. **c** Presentation of the transpose block, which is deployed in the decoder path for input upsampling and consists of a Minkowski convolution transpose layer, a Minkowski batch normalization layer, and a Minkowski ReLU activation function. **d** Depiction of the ResNet-inspired basic block, which possesses skip connections for effective feature extraction and is utilized in both the encoder and decoder paths

PUResNetV2.0, based on MCNNs, features an encoder-decoder framework [36] with 171 layers and 10,861,601 trainable parameters, tailored for binary segmentation, as illustrated in Fig. 2a. The architecture integrates an encoder and a decoder, both of which are constructed from multiple blocks. The encoder’s role is to reduce the dimensionality of the input—a SparseTensor depiction of a protein structure—by incorporating an assembly of convolution and basic blocks. The decoder, conversely, aims to upscale the encoder-produced feature maps. It uses a series of transpose and basic blocks, which are augmented by concatenating the corresponding feature maps from the skip pathway. These skip connections equip the decoder with detailed information from the encoder.

Figure 2b presents the convolution block, which is an integral part of the encoder pathway. The block incorporates a Minkowski convolutional layer [37], followed by a Minkowski batch normalization layer and a Minkowski ReLU activation function. Collectively, these components reduce the input dimensionality while simultaneously drawing out significant features. The combined use of Minkowski convolution, batch normalization, and ReLU activation empowers PUResNetV2.0 to learn complex input representations. Minkowski convolution layer inputs a 4-dimensional tensor, with three spatial dimensions (x,y,z) and one temporal dimension (t) and uses a hybrid kernel (non-hypercubic, non-permutohedral) of arbitrary shape for feature extraction [24]. The convolution operation in Minkowski convolution layer can be described with the equation below,

$$X_{u}^{out} = \mathop \sum \limits_{{i \in N^{D} \left( {u,C^{in} } \right)}} W_{i} x_{u + i}^{in} \;for\; u \in C^{out} ,$$

where N^D^ is a set of offsets that define the shape of a kernel and N^D^(u, C^in^) = {i|u + i ∈ C^in^, i ∈ N^D^} as the set of offsets from current center, u, that exists in C^in^. C^in^ and C^out^ are predefined input and output coordinates of sparse tensors. Minkowski convolution batch normalization layer and Minkowski ReLU activation function are adopted for sparse tensor from conventional batch normalization and ReLU activation function.

The decoder’s fundamental component, the transpose block, is visualized in Fig. 2c. It employs a Minkowski convolution transpose layer, Minkowski batch normalization, and a Minkowski ReLU activation function to upscale the input. These layers work in harmony to heighten the input’s spatial resolution and concurrently isolate pertinent features. The integration of Minkowski transposed convolution, Minkowski batch normalization, and Minkowski ReLU activation layers facilitates efficient upsampling, enhancing the model’s ability to distinguish between binding and non-binding atoms.

Figure 2d highlights the ResNet [38]-inspired basic block, which features skip connections between the input and output and is applied in both the encoder and decoder pathways of PUResNetV2.0. The deployment of the ResNet-derived basic block allows PUResNetV2.0 to effectively extract high-level and low-level features, effectively circumventing the issue of vanishing gradients.

### Optimizing PUResNetV2.0 using Optuna

To optimize PUResNetV2.0, we used Optuna, an automated hyperparameter optimization framework. We began the optimization process by defining the hyperparameter search space for PUResNetV2.0, incorporating parameters like batch size, learning rate, number of output planes and number of basic blocks. Our aim was to maximize the PRC AUC on the validation set, hence we established an objective function accordingly. Leveraging the Tree-structured Parzen Estimator (TPE), a Bayesian optimization algorithm, Optuna recommended hyperparameters by building a probabilistic model of the objective function. This sophisticated approach involves iteratively modeling and updating the probability distributions of hyperparameters to balance exploration and exploitation, ultimately guiding the search towards promising regions in the hyperparameter space with each iteration. We also incorporated an early stopping strategy to prevent overfitting, halting training if no improvement was seen in the validation loss over a predefined number of epochs.

### Postprocessing the predictions yielded by PUResNetV2.0

In the postprocessing phase of PUResNetV2.0, we utilized the density-based spatial clustering of applications with noise (DBSCAN) [39] algorithm, known for its advantage of not requiring a predetermined number of clusters, which was ideal for our scenario where the number of binding pockets was not known a priori. This algorithm took the xyz coordinates of the predicted binding atoms and grouped the proximate predictions, forming distinct binding pockets under the criterion of a minimum of five atoms within a spatial distance of 5.5 Å. Implementing the kd_tree algorithm and setting a leaf size of 100 enhanced its computational efficiency. This transformation of atomic-level predictions into identifiable binding pockets enables a more comprehensive analysis in protein-drug interaction studies.

### Performance benchmarking against PUResNetV1.0, DeepSurf, DeepSite, GRasP and P2Rank

To benchmark PUResNetV2.0’s performance, we extracted predictions from several established models, including PUResNetV1.0, DeepSurf, DeepSite, GRaSP, and P2Rank. For P2Rank and DeepSite, we obtained the predictions directly from the P2Rank datasets, which are available at https://github.com/rdk/p2rank-datasets. We implemented the models for PUResNetV1.0 and DeepSurf using their respective GitHub repositories (PUResNetV1.0: https://github.com/jivankandel/PUResNet; DeepSurf: https://github.com/stemylonas/DeepSurf) and subsequently generated predictions. For GRaSP, we sourced the predictions through its dedicated webserver at https://grasp.ufv.br/submit.

With P2Rank, each predicted binding pocket was ranked, and we chose the highest-ranked pocket for evaluation. We then calculated the pocket’s center based on its atomic coordinates. DeepSite provided the center for each predicted pocket, simplifying our extraction process. Similarly, DeepSurf also provided the predicted centers for the binding pockets. In the case of PUResNetV1.0, we used the atomic coordinates derived from the predicted pockets to calculate the center, facilitating standardization across the models for comparison purposes. Finally, with GRaSP, the predictions were supplied as residues within a CSV file, which we used to extract the necessary information for our analysis. Success rate based on DCA, Success rate based on DCC, precision, recall, F1 score, and MCC metrices are utilized to compare between methods.

A. Pocket centric method
  1. Success rate based on DCA
    DCA is the minimum distance between the center of predicted binding site to the any actual binding site atom. If the distance is ≤ 4 Å, then it its determined to be correctly predicted site and success rate is given by:
    $$Sucess\;Rate_{DCA} = \frac{{Number\;of\;predicted\;binding\;sites\;having \;DCA \le 4\,{\mathring{\text{A}}}}}{Total\;number\; of\; sites}.$$
  2. Success rate based on DCC
    DCC is the minimum distance between the center of predicted binding site to the center of the actual binding site. If the distance is ≤ 4Å, then it its determined to be correctly predicted site and success rate is given by:
    $$Sucess\;Rate_{DCC} = \frac{{Number\; of\; predicted\;binding\; sites\; having\;DCC \le 4\,{\mathring{\text{A}}}}}{Total\;number\;of\;sites}.$$
B. Residue centric method
  True Positive (TP) is correctly predicted residue as binding residues. False Positive (FP) is incorrectly predicted binding residues. True Negative (TN) is correctly predicted non-binding residue. False Negative (FN) is incorrectly predicted non-binding residue.
  1. Precision
    $Precision = \frac{{\mathop \sum \nolimits_{i = 0}^{n} \frac{{TP_{i} }}{{TP_{i} + FP_{i} }}}}{n}$ where n is the total number of protein structures.
  2. Recall
    $Recall = \frac{{\mathop \sum \nolimits_{i = 0}^{n} \frac{{TP_{i} }}{{TP_{i} + FN_{i} }}}}{n}$ where n is the total number of protein structures.
  3. F1 score
    $F1 \;score = \frac{{\mathop \sum \nolimits_{i = 0}^{n} \frac{{2 \times Precision_{i} \times Recall_{i} }}{{Precision_{i} + Recall_{i} }}}}{n}$ where n is the total number of protein structures.
  4. MCC
    $MCC = \frac{{\mathop \sum \nolimits_{i = 0}^{n} \frac{{TP_{i} \times TN_{i} - FP_{i} \times FN_{i} }}{{\sqrt {\left( {TP_{i} + FP_{i} } \right) \left( {TP_{i} + FN_{i} } \right)\left( {TN_{i} + FP_{i} } \right)\left( {TN_{i} + FN_{i} } \right)} }}}}{n}$ where n is the total number of protein structures.

### Implementation of the Web Server for PUResNetV2.0

To facilitate the application of PUResNetV2.0, we implemented a web server (https://nsclbio.jbnu.ac.kr/tools/jmol/) utilizing the Django Python web framework. The user interface was designed to provide options for uploading a PDB file or entering a PDB ID or UniProt ID. The platform also provides flexible preprocessing settings: a user-submitted protein structure can be processed as a single complex, individual chains can be treated as separate complexes, or selected chains (identified by a comma-separated list of their identifiers) can be treated as a single complex.

Once the necessary inputs and selections are provided by the user, the backend of the web server initiates the conversion of the protein structure into a Minkowski SparseTensor, as elaborated in “Data acquisition and processing” section. When the preprocessing setting is set to ‘single complex’, the given protein structure is converted into a single Minkowski SparseTensor. If ‘individual chains as separate complexes’ is selected, each chain is represented by an individual Minkowski SparseTensor, thus creating a batch. In scenarios where specific chains are selected, these chains are converted into a single Minkowski SparseTensor. Subsequently, the PUResNetV2.0 model is activated to generate predictions. The predicted binding atoms are then postprocessed by following the steps discussed in the “Postprocessing the Predictions Yielded by PUResNetV2.0” subsection, readying them for visualization.

In the final step, the predicted binding pockets are visually represented on the front-end side of the web server using the JSmol [40] Java-based viewer. This facilitates an interactive visualization of the 3D molecular structures of proteins and their predicted binding pockets, thereby providing the user with a graphical representation of the predictions. Furthermore, a list of the identified amino acids within each predicted binding pocket is also made available for download in the form of a PDB file. This comprehensive workflow from input processing to result visualization allows for a seamless and user-friendly experience on the web server, thereby maximizing the utility of PUResNetV2.0 for users.

## Results

### Diverse training dataset curated using a tailored parser

**Fig. 3:**
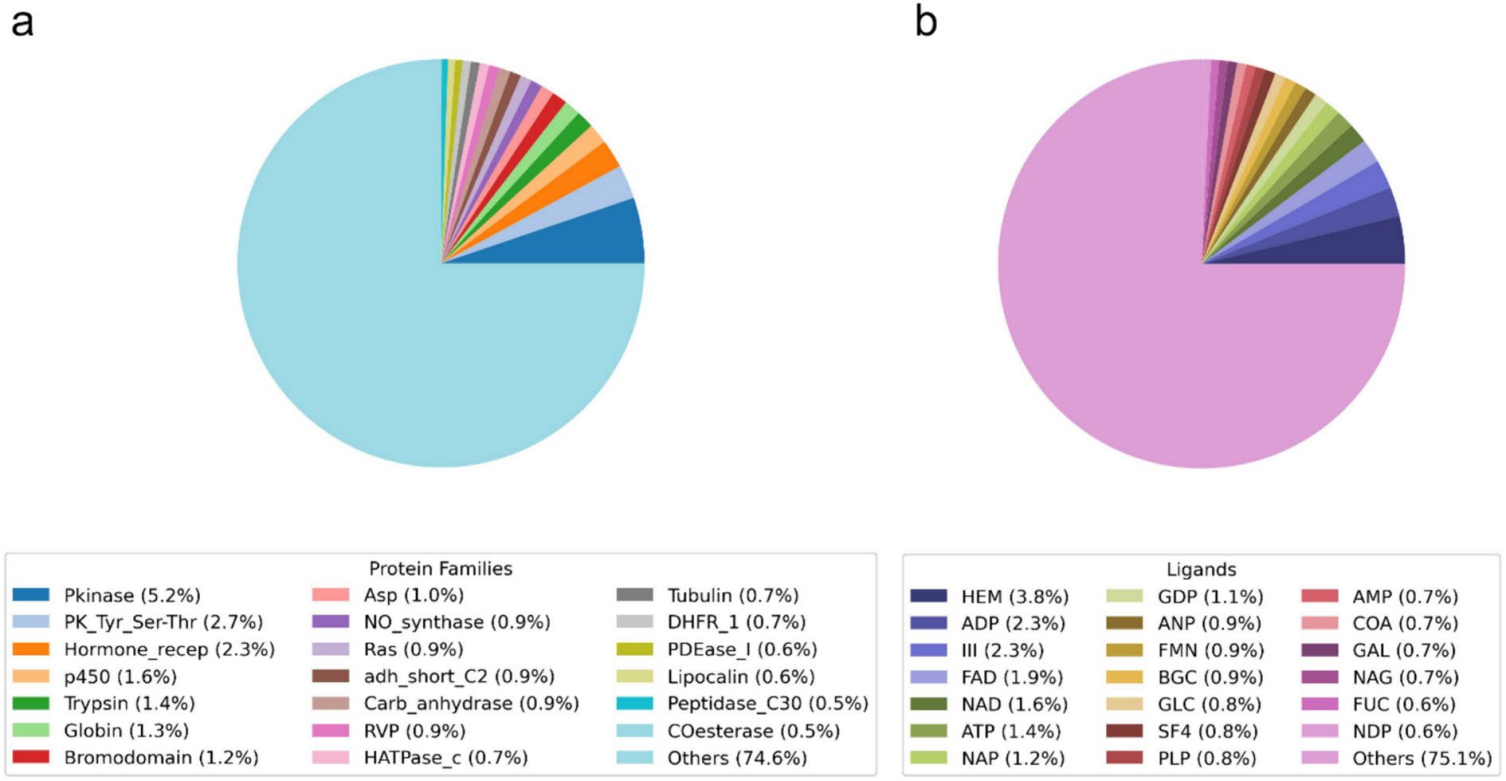
Distribution of the training dataset employed for PUResNetV2.0, categorized by its ligand types and protein families. These pie charts offer detailed insight into the breadth and depth of our dataset, encompassing 61,691 protein complexes. **a** Visualization of the distribution across 4729 protein families, emphasizing prevalent families such as Pkinase, PK_Tye_Ser-Thr, and Hormone_recep, which represent a significant proportion of the dataset. **b** Illustration of the diversity of 25,780 unique ligand binding sites included in the dataset, pointing out commonly found ligand binding site such as HEM, ADP, and III

In the initial phase of our research, we curated a dataset crucial for the development of the PUResNetV2.0 model, with a focus on protein–ligand interaction sites. This dataset comprised 61,691 protein complexes, encompassing 25,780 unique ligand-binding sites across 4729 protein families. We extracted PDB and Ligand IDs from the BioLip database and downloaded the corresponding structures from the RCSB database. To concentrate on small molecule ligand-binding sites, we excluded binding sites associated with ions, water molecules, small peptides, and polynucleotides. Moreover, we removed the HETATM records from each PDB file in the dataset preparation phase. This methodology offered a detailed perspective on vital ligand-binding sites. For example, among the protein complexes, 3.8% contained a HEM binding site, 2.3% an ADP binding site, and another 2.3% an III binding site. In terms of protein families, 5.2% of the complexes were from the Pkinase family, 2.7% from the PK_Tyr_Ser-Thr family, and 2.3% from the Hormone_recep family, illustrating the dataset’s diversity as shown in Fig. 3.

While preparing our dataset, we acknowledged the inherent imbalance that characterizes real-world protein‒ligand interactions. Specifically, atoms involved in interactions are far outnumbered by those that do not partake in such interactions. To ensure a fair model performance evaluation and eliminate data leakage, we implemented an 80/20 split for the training and validation sets, ensuring that protein complexes interacting with the same ligand were exclusive to either set. This careful dataset preparation and division process laid the foundation for the successful training and optimization of our PUResNetV2.0 model.

To aid in the development of our dataset, we created a custom parser specifically designed for converting given PDB files to Minkowski SparseTensor representations. Our parser, based on the specifications provided by the WorldWide Protein Data Bank (wwPDB), parsed the atomic records from the associated PDB files. One of the key features of our parser is its capability to directly access and process protein structures from the RCSB database, streamlining the data input process for users and facilitating the efficient preparation of data for PUResNetV2.0 training and evaluation. More detailed usage examples are available in our GitHub repository.

The PUResNetV2.0 optimization process improved the validation PRC AUC from 46 to 71%.

In the quest to improve our model’s performance on the highly imbalanced dataset, we initiated our optimization process using the Optuna library. The weighted adaptive moment estimation (AdamW) optimizer [41] was used as the optimizer. Initially, we used the Dice loss function [42] as a loss function due to its effectiveness in balancing the contributions of the foreground (atoms involved in interactions) and the background (atoms not involved in interactions) by considering both precision and recall in its calculation. Despite its well-regarded ability to manage disparities between classes, the application of the Dice loss function led to a validation area under the precision recall curve (PRC AUC) of 46% and an F1 score of 61%, as shown in Table 1, indicating that further optimization was required to enhance the performance of our PUResNetV2.0 model.

**Table 1:** Hyperparameter optimization results obtained for PUResNetV2.0

	Batch size	LR	Loss function	Out planes		Number of basic blocks	Validation	
				Encoder basic blocks	Transpose blocks		PRC AUC (%)	F1 score (%)
a)	80	7.89e^−4^	Focal loss (γ = 1, α = 0.15)	32, 48, 128, 128	128, 128, 48, 32	2, 3, 1, 3, 3, 2, 1, 3	71	65
b)	96	4.21e^−4^	Focal loss (γ = 2, α = 0.10)	32, 48, 128, 128	128, 128, 48, 32	2, 2, 1, 3, 1, 2, 1, 1	70	64
c)	64	1.65e^−3^	Dice loss	32, 32, 112, 80	80, 112, 32, 32	3, 1, 1, 3, 1, 2, 1, 1	46	61
d)	128	1.03e^−4^	Dice loss	32, 48, 96, 128	128, 96, 48, 32	2, 3, 3, 3, 1, 2, 2, 1	45	60

The loss graphs labeled as a, b, c, and d correspond to the respective hyperparameter configurations delineated in Table 1. Each graph presents a comparative analysis of training vs validation loss, substantiating the assertion that the model exhibits neither overfitting nor underfitting.

**Fig. 4:**
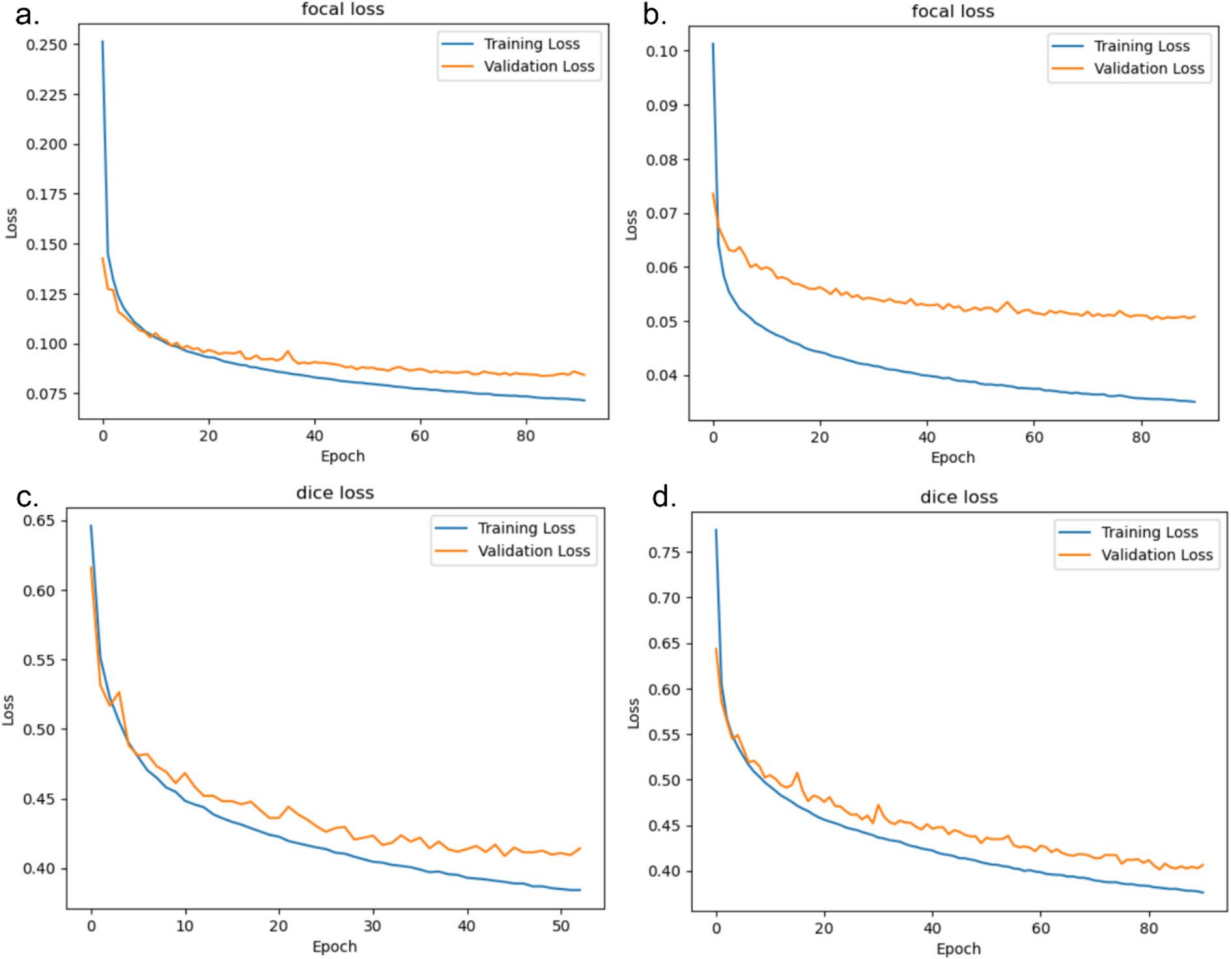
Training loss vs validation loss for each configuration in Table 1

In response, we pivoted our approach to focus on the focal loss function [43]. Noted for its capacity to handle imbalanced datasets by concentrating on challenging examples, the implementation of the focal loss function served as the turning point in our model’s optimization process. Through rigorous hyperparameter tuning performed using the Optuna library, we observed a significant leap in our model’s predictive ability, with the validation PRC AUC of our optimized PUResNetV2.0 model reaching an impressive 71% and its F1 score improving to 65%, as shown in Table 1. As shown in Fig. 4, the graph shows for each hyperparameter configuration, the model is stable and well-tuned. Using the best hyperparameters identified, a 10-fold cross-validation was performed. The results, as detailed in Table 2, showed an average PRC AUC of 70.00% with a standard deviation of 0.011%, and an F1 score of 64.03% with a standard deviation of 0.016%, underscoring the model’s steady performance across different data segments. This substantial improvement in the validation PRC AUC underscores the effectiveness of the focal loss function in cases with highly imbalanced protein‒ligand interaction data. Notably, the high validation PRC AUC score indicates the ability of PUResNetV2.0 to correctly predict the atoms involved in interactions.

**Table 2:** 10-fold cross validation results

Experiment	Training sample	Validation sample	Validation PRC AUC	Validation F1 score
1	55,521	6170	69.14%	62.27%
2	55,522	6169	70.70%	64.10%
3	55,522	6169	68.70%	62.90%
4	55,522	6169	70.40%	64.40%
5	55,522	6169	70.20%	64.50%
6	55,522	6169	70.20%	63.80%
7	55,522	6169	71.90%	67.80%
8	55,522	6169	68.00%	62.10%
9	55,522	6169	71.00%	65.10%
10	55,522	6169	69.80%	63.30%
Average			70.00%	64.03%
Standard deviation			0.011455	0.0164673
Variance			0.0001312	0.0002712

### PUResNetV2.0 identifies binding pockets of complex protein structures

Our examination of PUResNetV2.0’s prediction results obtained across the Holo801 and Apoholo45 datasets elucidated the model’s capability to navigate the intricacies of protein structures. Evidently, the model’s predictions varied significantly depending on the context in which we presented the protein structures: as individual chains each forming separate complexes, as groups of two or more chains that constituted a larger complex, or as an integrated structure treated as a single complex.

**Fig. 5:**
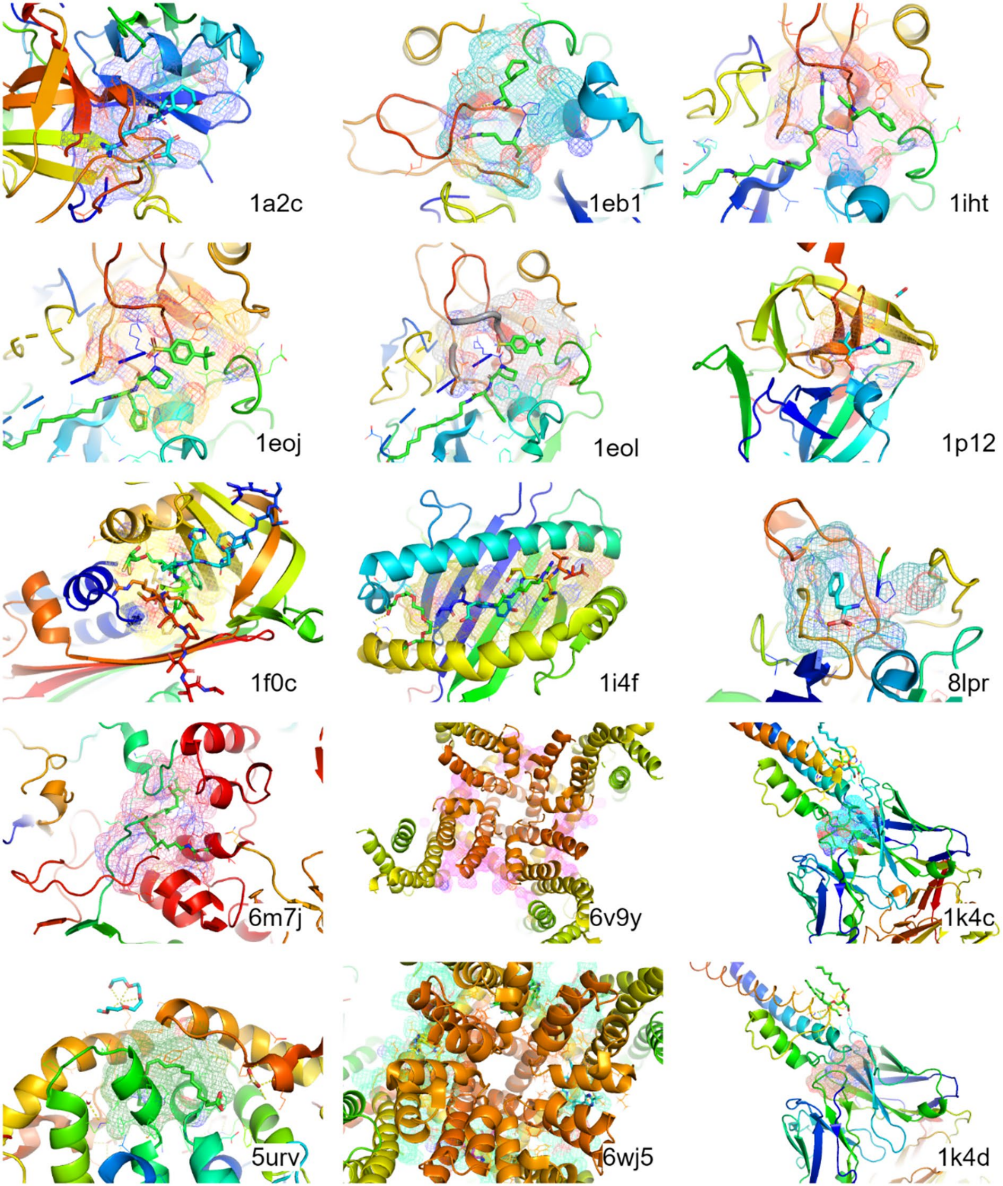
Visual representation of PUResNetV2.0’s capabilities in terms of accurately predicting diverse protein‒ligand binding sites across a variety of complex protein structures. The structures of proteins are displayed in cartoon format, with the corresponding ligands represented by stick models. The meshes overlaid on these structures signify the predicted binding pockets as determined by PUResNetV2.0. Each protein structure’s PDB ID is provided at the bottom right side of the structure. This figure illustrates the model’s competence in identifying potential binding sites across a range of protein‒ligand complexes

A noteworthy aspect of our analysis was the model’s approach for handling the protein structures in the Holo801 dataset that incorporated peptide-like ligands, specifically structures 1a2c, 8lpr, 1eoj, 1eol, 1i4f, 1eb1, 1p12, 1iht, and 1f0c, as shown in Fig. 5. As these peptide-like ligands were not represented in our training dataset, they introduced an element of novelty to the test scenario. If we presented the peptide-containing structures as a unified complex, the model refrained from providing predictions. In contrast, when we interpreted these structures as a complex excluding the peptides, PUResNetV2.0 successfully discerned the binding pockets for these peptide-like ligands. Additionally, in the cases with antibody-antigen complexes 1k4c and 1k4d from the Holo801 dataset shown in Fig. 5, when considering the heavy and light chains as a complex, PUResNetV2.0 successfully predicted the binding region where the antigen and antibody bound.

Additional remarkable insights were derived from the analysis conducted on the Apoholo45 dataset, more specifically, structures 6m7j, 6v9y, 6wj5, and 5urv depicted in Fig. 5. For instance, when we treated chains C and D of the bounded structure 6m7j as a larger complex, PUResNetV2.0 accurately identified the binding pocket for the COL ligand, which interacted with both chains. Additionally, when treating structures 6v9y, 6wj5, and 5urv as a single complex, PUResNetV2.0 precisely predicted the binding pockets.

In conclusion, the performance of PUResNetV2.0 in accurately identifying binding pockets across various scenarios and structures, including the nuanced complexities of structures with peptide-like ligands and antigen–antibody complexes in the Holo801 dataset and the ligand interactions across multiple chains in the Apoholo45 dataset, exhibited PUResNetV2.0’s versatility and adaptability, positioning it as a powerful tool in the field of protein structure analysis and ligand binding site prediction.

### Comparative benchmark analysis reveals PUResNetV2.0’s better performance

In this study, we evaluated the performance of PUResNetV2.0, our proposed LBSP model, against established methods such as P2Rank, DeepSurf, PUResNetV1.0, DeepSite, and GRaSP. This analysis used four distinctive benchmark datasets, Coach100, Holo801, Apoholo45 and PDBBind1681, each offering unique challenges. Coach100, comprising only monomeric protein structures, assessed the models’ proficiency in handling simpler, individual protein structures. Conversely, Holo801, laden with ligand-bound oligomeric structures, tested the models’ abilities to interpret complex interactions across multiple protein chains. The PDBBind1681 is a high-quality dataset originally used for developing and validating scoring functions and docking methods which contains bindings residues information for each target protein. The Apoholo45 dataset stood out by incorporating both ‘apo’ and ‘holo’ protein structures, pushing the models to discern and differentiate between these critical states for accurate ligand binding site prediction.

A comparison of the model performances achieved across the benchmark datasets revealed insightful trends. The performance of various models on the Coach100 dataset, characterized by simpler monomeric structures and ion binding sites, was generally lower, with DeepSite exhibiting the poorest results among them. Conversely, when tested against the Holo801 dataset, composed of complex oligomeric structures, most models showed higher success rates, except for PUResNetV1.0, which exhibited a significant drop, indicating its challenges in managing such complex structures. The Apoholo45 dataset, which comprises both ‘apo’ and ‘holo’ protein structures, presented an added challenge that led most models, including DeepSurf and GRaSP, to struggle.

PUResNetV2.0 consistently demonstrated improved performance over the P2Rank, DeepSurf, PUResNetV1.0 and DeepSite methods by attaining elevated DCA and DCC success rates across the Coach100, Holo801, and Apoholo45 benchmark datasets, as highlighted in Table 3. In the case of the Coach100 dataset, PUResNetV2.0 achieved 59.0% DCA and 38.0% DCC success rates. Regarding the Holo801 dataset, PUResNetV2.0 yielded 85.4% DCA and 53.7% DCC success rates. Finally, for the Apoholo45 dataset, PUResNetV2.0 attained 71.1% DCA and 40.0% DCC success rates.

**Table 3:** Comparison among the performances of P2Rank, DeepSurf, PUResNetV1.0, DeepSite, and PUResNetV2.0 on benchmark datasets

Benchmark dataset	Methods	Success rate (DCA ≤ 4Å) (%)	Success rate (DCC ≤ 4Å) (%)
Coach100	P2Rank	44.0	29.0
	DeepSurf	51.0	26.0
	PUResNetV1.0	51.0	**38.0**
	DeepSite	27.0	15.0
	PUResNetV2.0	**59.0**	**38.0**
Holo801	P2Rank	74.0	48.2
	DeepSurf	83.8	46.5
	PUResNetV1.0	2	0.9
	DeepSite	71.4	33.8
	PUResNetV2.0	**85.4**	**53.7**
Apoholo45	DeepSurf	46.7	16.7
	PUResNetV2.0	**71.1**	**40.0**

Top values for each benchmark dataset are represented in bold

As shown in Table 4, compared to GRaSP, the PUResNetV2.0 method exhibited substantial performance enhancements on the Coach100, Holo801, Apoholo45 and PDBBind1681 datasets, particularly in terms of the F1 score, MCC, and recall metrics. Nevertheless, GRaSP excelled with respect to precision on the Coach100. In the case of PDBBind1681 dataset, PUResNetV2.0 notably outperforms GRaSP, as evidenced by its enhanced metrics, achieving a precision of 78.68%, recall of 35.90%, F1 score of 47.10%, and a MCC of 46.70%. In summary, PUResNetV2.0 consistently yielded superior results for most metrics, achieving a minimum MCC increase of 10% and a 10% F1 score improvement over GRaSP.

**Table 4:** Comprehensive assessment of PUResNetV2.0 and GRaSP on benchmark datasets

Benchmark dataset	Methods	Precision (%)	Recall (%)	F1 score (%)	MCC (%)
Coach100	PUResNetV2.0	61.3	**62.4**	**61.8**	**62.4**
	GRaSP	**63.5**	43.9	51.9	50.1
Holo801	PUResNetV2.0	**70.2**	**71.0**	**70.6**	**68.3**
	GRaSP	66.9	50.1	57.3	55.1
Apoholo45	PUResNetV2.0	**69.7**	**54.2**	**61.0**	**59.4**
	GRaSP	61.7	32.0	42.1	42.0
PDBBind1681	PUResNetV2.0	**78.68**	**35.90**	**47.10**	**46.70**
	GRaSP	57.31	22.89	31.01	30.84

Top values for each benchmark dataset are represented in bold

## Discussion

The insights garnered from this research have demonstrated the remarkable potential of PUResNetV2.0 for accomplishing the challenging task of LBSP. Through the careful curation of a comprehensive dataset encompassing a wide range of protein complexes and the optimization of PUResNetV2.0, we achieved a remarkable improvement in the validation PRC AUC from 46 to 71% in the presence of a highly imbalanced dataset. The key findings from our study revealed the ability of PUResNetV2.0 to adeptly predict binding pocket, especially for complex structures housing peptide-like ligand. Additionally, it consistently outperformed other methods across benchmark datasets, including Holo801, Coach100, Apoholo45, and PDBBind1681. These findings, characterized by PUResNetV2.0’s enhanced performance not only underscore the significant strides our study has made in the field of LBSP but also set the stage for an in-depth exploration of our findings, their implications, and potential avenues for future research.

The transition from PUResNetV1.0 to PUResNetV2.0 represents an important journey of continuous evolution in protein structure representation and feature extraction for LBSP. A critical insight gained during this process is the importance of the quality and diversity of the utilized training dataset in driving the predictive power and generalizability of the resulting model. Both versions are rooted in the robust UNet [36] and ResNet [38] architectures, yet their feature extraction methods differ: PUResNetV1.0 utilizes 3D CNNs, while PUResNetV2.0 adopts MCNNs. MCNNs are specifically designed for the efficient processing and extraction of features from sparse representations. However, transitioning to MCNNs and sparse representations was not sufficient for ensuring success; we also needed to address the imbalance issues that are frequently found in the datasets of this field. In PUResNetV1.0, we managed these issues with the Dice loss, a strategy carried forward to PUResNetV2.0. However, the Dice loss only yielded a 46% PRC AUC on our validation dataset. Upon switching to focal loss, our performance improved significantly, achieving a 71% PRC AUC on the validation dataset. This stark difference emphasizes the need for utilizing appropriate loss functions when handling imbalanced data, leading to our model’s improved performance.

PUResNetV1.0 was trained on the 2017 version of the sc-PDB dataset [34]. Although comprehensive for its time, we found this dataset to be limited in terms of representing the diversity and complexity of protein structures, particularly oligomeric structures, which directly impacted the performance of PUResNetV1.0. PUResNetV1.0 had difficulties dealing with the Holo801 dataset, which is composed of complex oligomeric structures. However, it was more adept at handling the monomeric structures in the Coach100 dataset. The shortcomings of PUResNetV1.0 pushed us toward a more advanced approach for PUResNetV2.0, adopting a sparse representation method inspired by Minkowski SparseTensor’s application in LiDAR segmentation.

The proposed method outperformed existing ones across a variety of datasets, including Coach100, Holo801, Apoholo45 and PDBBind1681. The superiority of PUResNetV2.0 can be partially attributed to its ability to adeptly avoid the errors observed in other models. For example, while treating oligomeric structures as surface representations of a protein with a set of local 3D voxelized grids placed on the protein’s surface, DeepSurf introduced errors in its predictions. DeepSurf identified residues of peptide-like ligands as binding residues (as shown in Additional file Table 1). A similar inability was observed with the machine learning based P2Rank method, while GRasP simply failed to process such structures. In contrast, PUResNetV2.0, leveraging the advantages of Minkowski SparseTensors to represent protein structures, was able to process such structures as well as restrained to predict residues of peptide-like ligands as binding residues as evidenced in “PUResNetV2.0 Identifies Binding Pockets of Complex Protein Structures”. This reflects the potential of sparse representation in LBSP, not only in terms of improving performance but also in advancing the field of LBSP, especially in scenarios involving complex molecular interactions.

While PUResNetV2.0 has demonstrated a significant advancement over its predecessor and other methodologies, it does not come without its own limitations. A primary constraint is that the model’s performance is contingent upon the input training dataset. Currently, our dataset does not account for all types of binding sites, with notable exclusions being ions, DNA/RNA, and peptide-like ligand binding sites. These ligand types demonstrate unique interaction patterns with proteins. For example, ions typically interact with proteins through salt bridges or coordinate bonds. Interactions between DNA/RNA and proteins typically engage larger surface areas, commonly occurring in the grooves or channels of the protein. Peptide-like ligands present a spectrum of interaction patterns, which are largely dependent on their lengths and sequences. In addition, our present training dataset primarily encompasses orthosteric sites, neglecting allosteric sites that play a vital role in protein‒ligand interactions. The omission of these entities constitutes a significant limitation, as their unique interaction patterns can substantially influence the precision and applicability of our model’s predictions. Consequently, PUResNetV2.0 in its current form may lack effectiveness in terms of predicting binding sites that involve these omitted ligand types.

Addressing these limitations necessitates a more nuanced approach in future research. An integral part of this approach would be the expansion of the training dataset to include more diverse types of binding sites, especially those involving ions, DNA/RNA, and peptide-like ligands. Given their distinct interaction patterns with proteins, these ligand types could benefit from dedicated models trained on specialized datasets curated specifically for each ligand type. Our custom parser, developed to convert protein structures into Minkowski SparseTensor representations, provides a robust tool for streamlining the curation of these specialized datasets. However, we must not overlook the complexity that accompanies this approach. Developing and validating separate models for each ligand type could pose significant challenges, particularly in maintaining the balance between specialization and generalizability. This task also demands the careful tuning of model parameters and loss functions for each ligand-specific model. Nevertheless, the potential rewards—improved accuracy, broader applicability, and greater insights into unique interaction patterns—make this a promising direction for future research.

## Conclusion

In conclusion, this study combines well-curated training datasets, innovative protein structure representations via Minkowski SparseTensors, and a strategically selected loss function, all geared toward addressing the intricate challenges of LBSP. The transition from dense to sparse data representations has significantly elevated PUResNetV2.0’s ability to manage complex protein structures, outperforming previous methods across diverse datasets. Although some areas demand further refinement, specifically the representation of the complete range of protein‒ligand interactions, the potential of PUResNetV2.0 in facilitating the drug discovery process, coupled with our user-friendly web server, stands as a significant achievement. This account highlights the strides we have made thus far. As we continue to refine our methodologies and broaden our training datasets, we expect to uncover deeper insights and achieve even higher levels of accuracy and inclusivity in predicting protein‒ligand interactions, propelling the field of LBSP to new heights.

### Supplementary Information

Supplementary Material 1. Comparative binding site predictions yielded by PUResNetV2.0, GRasP, DeepSurf, and P2Rank for protein structures containing peptide-like ligands.

Supplementary Material 2. Documentation for the PUResNetV2.0 Webserver.

## Data Availability

The associated results and training data are available for public access and can be obtained from our dedicated repository at https://nsclbio.jbnu.ac.kr/pv2_dataset/. The repository comprises several files—Training.csv: this file contains information about the training data. Training_data.zip: this archive includes the processed data that were employed for training purposes—(i) pv2_results: this folder encompasses all the evaluation results, which have been further categorized into the following files. (ii) Coach100_bench.csv: this file presents the results obtained on the Coach100 dataset, including DCC and DCA values. (iii) Holo801_bench.csv: this file provides the results obtained on the Holo801 dataset, encompassing DCC and DCA values. (iv) apoholo_bench.csv: this file documents the results obtained on the Apoholo45 dataset, featuring DCC and DCA values. (v) pv2gasp.zip: this archive contains the combined results of GRaSP and our PUResNetV2.0 method.

## Abbreviations

LBSP: Ligand binding site prediction
DCA: Distance center atom
DCC: Distance center center
MCC: Matthews correlation coefficient
PRC AUC: Precision–recall curve area under the curve
PDB: Protein Data Bank
RNA: Ribonucleic acid
DNA: Deoxyribonucleic acid
RCSB: Research collaboratory for structural bioinformatics
SAS: Solvent accessible surface
CNN: Convolutional neural network
MCNN: Minkowski convolutional neural network
DBSCAN: Density-based spatial clustering of applications with noise
wwPDB: Worldwide Protein Data Bank
SDF: Structure data file
LiDAR: Light detection and ranging
AdamW: Adaptive moment estimation with weight decay
DCNN: Deep convolutional neural network
TPE: Tree-structured Parzen estimator

## Materials availability

Our webserver can be accessed at https://nsclbio.jbnu.ac.kr/tools/jmol. Detailed documentation is provided in the Supplementary Information as well as in the webserver to assist with navigation and usage.

## Code availability

The code for this project is publicly accessible and can be found at https://github.com/jivankandel/PUResNetV2.0.git. It comes with comprehensive documentation for ease of understanding and utilization.
