# Supplementary material for: PUResNetV2.0: a deep learning model leveraging sparse representation for improved ligand binding site prediction
Source: J Cheminform. 2024 Jun 7;16:66. doi: 10.1186/s13321-024-00865-6 (PMC11157904; doi:10.1186/s13321-024-00865-6)
Supplement: Supplementary file 1 — Supplementary Material 1. Comparative binding site predictions yielded by PUResNetV2.0, GRasP, DeepSurf, and P2Rank for protein structures containing peptide-like ligands. [file 13321_2024_865_MOESM1_ESM.docx]

PUResNetV2.0: A Deep Learning Model Leveraging Sparse Representation for Improved Ligand Binding Site Prediction

Kandel Jeevan^1,+^, Shrestha Palistha^2,+^, Hilal Tayara^3,*^, and Kil T. Chong^1,2,3,4,*^

^1^Graduate School of Integrated Energy-AI, Jeonbuk National University, Jeonju, 54896, South Korea

^2^Department of Electronics and Information Engineering, Jeonbuk National University, Jeonju, 54896, South Korea

^3^School of International Engineering and Science, Jeonbuk National University, Jeonju, 54896, South Korea

^4^Advanced Electronics and Information Research Center, Jeonbuk National University, Jeonju, 54896, South Korea

^*^hilaltayara@jbnu.ac.kr, kitchong@jbnu.ac.kr

^+^these authors contributed equally to this work

# List of Tables

[Table 1: Comparative binding site predictions yielded by PUResNetV2.0, GRasP, DeepSurf, and P2Rank for protein structures containing peptide-like ligands. Residues highlighted in red correspond to peptide-like ligand residues that were inaccurately predicted. 3](#_Toc159945053)

Table 1: Comparative binding site predictions yielded by PUResNetV2.0, GRasP, DeepSurf, and P2Rank for protein structures containing peptide-like ligands. Residues highlighted in red correspond to peptide-like ligand residues that were inaccurately predicted.

| PDB | Ligand | GRasP | PUResNetV2.0 | DeepSurf | P2Rank |
| --- | --- | --- | --- | --- | --- |
| 1a2c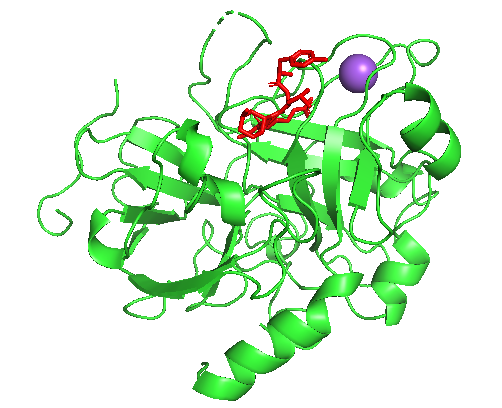 | AERUGINOSIN  298-A | NA | HIS:57:H TRP:60D:H TYR:60A:H GLU:97A:H ASN:98:H LEU:99:H ILE:174:H ASP:189:H ALA:190:H CYS:191:H GLU:192:H GLY:193:H SER:195:H VAL:213:H SER:214:H TRP:215:H GLY:216:H GLU:217:H GLY:219:H CYS:220:H ASP:221:H GLY:226:H PHE:227:H | HIS:57:H  CYS:58:H  TYR:60A:H  GLU:97A:H  LEU:99:H  ASP:189:H  ALA:190:H  CYS:191:H  GLU:192:H  SER:195:H  VAL:213:H  TRP:215:H  GLY:216:H  GLY:219:H  CYS:220:H  PHE:227:H  LEU:2:J | H:192  H:193  H:195  H:41  H:42  H:57  H:60D H:60F H:60H  J:3 |
| 8lpr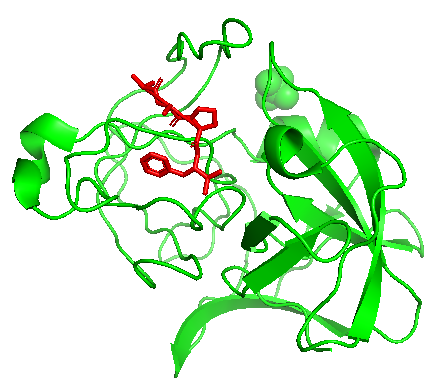 | O-methylsuccinyl-alanyl-alanyl-prolyl-borophenylalanine | NA | HIS:57:A TYR:171:A GLU:174:A ARG:192B:A MET:192:A GLY:192A:A GLY:193:A ASP:194:A SER:195:A SER:214:A GLY:215:A GLY:216:A ASN:217:A GLY:217E:A VAL:217A:A ASN:218:A ASN:219:A | GLY:140:A  MET:192:A  GLY:192A:A  ARG:192B:A  SER:195:A  GLY:216:A  VAL:217A:A  ASN:219:A  ALA:3:P  PRO:2:P | A:192B A:193  A:195  A:40  A:41  A:42  A:57  A:58  P:2 |
| 1eoj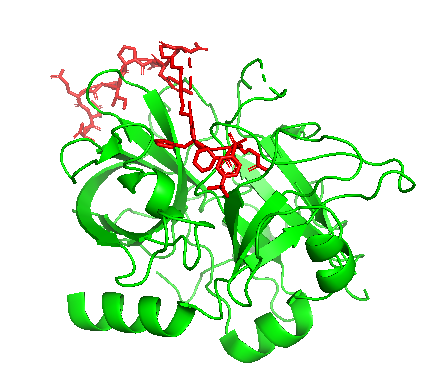 | THROMBIN INHIBITOR P798 | NA | HIS:57:A TRP:60D:A TYR:60A:A GLU:97A:A ASN:98:A LEU:99:A ILE:174:A ASP:189:A ALA:190:A CYS:191:A GLU:192:A GLY:193:A SER:195:A VAL:213:A SER:214:A TRP:215:A GLY:216:A GLU:217:A GLY:219:A CYS:220:A GLY:226:A | LEU:41:A  CYS:42:A  HIS:57:A  CYS:58:A  TYR:60A:A  TRP:60D:A  LYS:60F:A  PHE:60H:A  LEU:99:A  SER:195:A  SER:214:A  TRP:215:A  ARG:2:B | A:174  A:215  A:60A A:60C A:60D  A:96  A:97  A:97A  A:98  A:99  B:2  B:3 |
| 1eol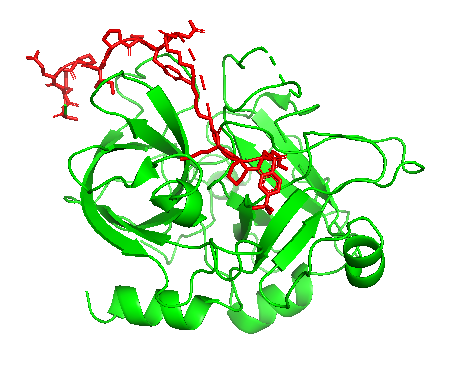 | THROMBIN INHIBITOR P628 | NA | HIS:57:A TRP:60D:A TYR:60A:A TRP:96:A GLU:97A:A ASN:98:A LEU:99:A ILE:174:A ASP:189:A ALA:190:A CYS:191:A GLU:192:A GLY:193:A SER:195:A VAL:213:A SER:214:A TRP:215:A GLY:216:A GLU:217:A GLY:219:A CYS:220:A GLY:226:A | LEU:41:A  HIS:57:A  TYR:60A:A  TRP:60D:A  LYS:60F:A  LEU:99:A  GLY:193:A  SER:195:A  SER:214:A  TRP:215:A  ARG:2:B | A:174  A:215  A:60A A:60C A:60D  A:96  A:97  A:97A  A:98  A:99  B:2  B:3 |
| 1i4f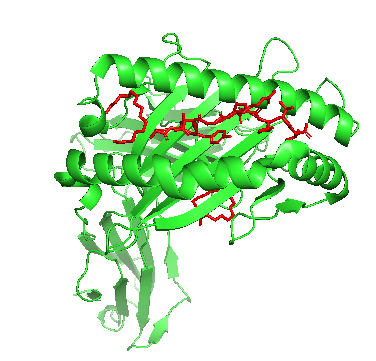 | MELANOMA-ASSOCIATED ANTIGEN 4, PEG | TYR:7:A  TYR:99:A  TRP:147:A  TRP:167:A  TRP:171:A  HIS:31:B | MET:5:A TYR:7:A PHE:9:A MET:45:A TYR:59:A GLU:63:A LYS:66:A VAL:67:A HIS:70:A THR:73:A ASP:77:A THR:80:A LEU:81:A TYR:84:A ARG:97:A TYR:99:A HIS:114:A TYR:116:A TYR:123:A THR:143:A LYS:146:A TRP:147:A VAL:152:A GLN:155:A LEU:156:A TYR:159:A THR:163:A TRP:167:A TYR:171:A | ARG:6:A  PHE:8:A  TYR:27:A  THR:31:A  GLN:32:A  AGLN:32:A  BGLN:32:A  ARG:181:A  PRO:235:A  GLY:237:A  ASP:238:A  GLY:239:A  PHE:241:A  TYR:26:B  ILE:35:B  SER:52:B  LEU:54:B  PHE:56:B  SER:57:B  TYR:63:B  LEU:64:B  LEU:65:B  TYR:67:B | A:113  A:211  A:232  A:233  A:235  A:237  A:238  A:239  A:241  A:27  A:29  A:30  A:31  A:32  A:48  A:6  A:8  A:98  B:26  B:28  B:29  B:51  B:52  B:53  B:55  B:56  B:57  B:58  B:59  B:6  B:61  B:63  B:65  B:67 |
| 1eb1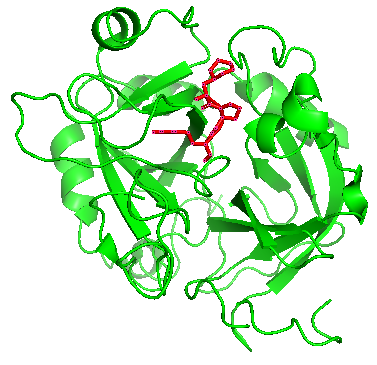 | 3-CYCLOHEXYL-D-ALANYL-L-PROLYL-N~2~-METHYL-L-ARGININE | NA | HIS:57:H TRP:60D:H LYS:60F:H TYR:60A:H GLU:97A:H ASN:98:H LEU:99:H ILE:174:H ASP:189:H ALA:190:H CYS:191:H GLU:192:H GLY:193:H ASP:194:H SER:195:H VAL:213:H SER:214:H TRP:215:H GLY:216:H GLU:217:H GLY:219:H CYS:220:H GLY:226:H PHE:227:H TYR:228:H | GLU:146:H  ASP:189:H  ALA:190:H  CYS:191:H  GLU:192:H  GLY:193:H  ASP:194:H  SER:195:H  TRP:215:H  GLY:216:H  GLY:219:H  CYS:220:H  GLY:226:H  TYR:228:H  PRO:2:B | H:192  H:193  H:39  H:40  H:41  H:57  H:60D H:60F H:60H |
| 1p12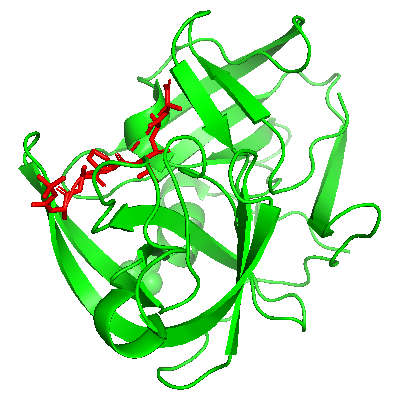 | PHOSPHONATE ESTER INHIBITOR |  | HIS:57:E PHE:94:E TYR:171:E GLU:174:E ARG:192B:E GLY:192A:E MET:192:E GLY:193:E ASP:194:E SER:195:E MET:213:E SER:214:E GLY:215:E GLY:216:E VAL:217A:E | CYS:58:E  GLY:120B:E  ASER:120D:E  BSER:120D:E  SER:139:E  ARG:141:E  SER:195:E  SER:214:E  GLY:216:E  PRO:2:I | E:192A E:192B E:193  E:195  E:215  E:42  E:57  E:58  I:2 |
| 1iht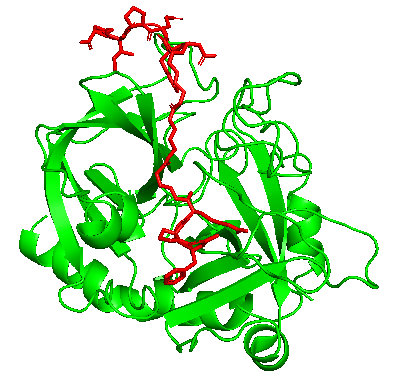 | HIRUTONIN-6 | NA | HIS:57:H TRP:60D:H TYR:60A:H GLU:97A:H ASN:98:H LEU:99:H ILE:174:H ASP:189:H ALA:190:H CYS:191:H GLU:192:H GLY:193:H ASP:194:H SER:195:H VAL:213:H SER:214:H TRP:215:H GLY:216:H GLU:217:H GLY:219:H CYS:220:H TYR:225:H GLY:226:H PHE:227:H | HIS:57:H  ASP:189:H  ALA:190:H  CYS:191:H  GLU:192:H  GLY:193:H  SER:195:H  VAL:213:H  SER:214:H  TRP:215:H  GLY:216:H  GLY:219:H  CYS:220:H  GLY:226:H  PHE:227:H  TYR:228:H  PRO:2:I | H:192  H:193  H:195  H:40  H:41  H:42  H:57  H:60D H:60F  I:2 |
| 1f0c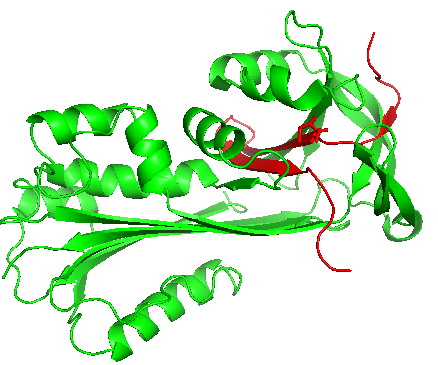 | ICE INHIBITOR, DTT | NA | MET:35:A PHE:38:A ASN:49:A VAL:50:A PHE:51:A ILE:52:A SER:53:A PRO:54:A PRO:55:A PHE:112:A VAL:188:A PHE:190:A ILE:240:A SER:249:A MET:250:A VAL:251:A VAL:252:A ILE:253:A LEU:254:A LEU:260:A ILE:263:A GLU:264:A LEU:267:A PHE:272:A ILE:288:A PHE:291:A ILE:338:A ALA:347:A | LEU:173:A  ASP:174:A  ALA:331:A  MET:332:A  ILE:333:A  CYS:352:A | A:210 A:257A A:258  A:260  A:261  A:264  A:46  A:47  B:369  B:387  B:389 |
