# Supplementary material for: PUResNetV2.0: a deep learning model leveraging sparse representation for improved ligand binding site prediction
Source: J Cheminform. 2024 Jun 7;16:66. doi: 10.1186/s13321-024-00865-6 (PMC11157904; doi:10.1186/s13321-024-00865-6)
Supplement: Supplementary file 2 — Supplementary Material 2. Documentation for the PUResNetV2.0 Webserver. [file 13321_2024_865_MOESM2_ESM.docx]

**Documentation for the PUResNetV2.0 Webserver**

The PUResNetV2.0 webserver is a powerful tool designed to offer comprehensive protein binding pocket prediction and visualization capabilities. The platform is built on Django, ensuring a user-friendly interface, and streamlined operations for molecular biology researchers.

# Webserver Implementation

**
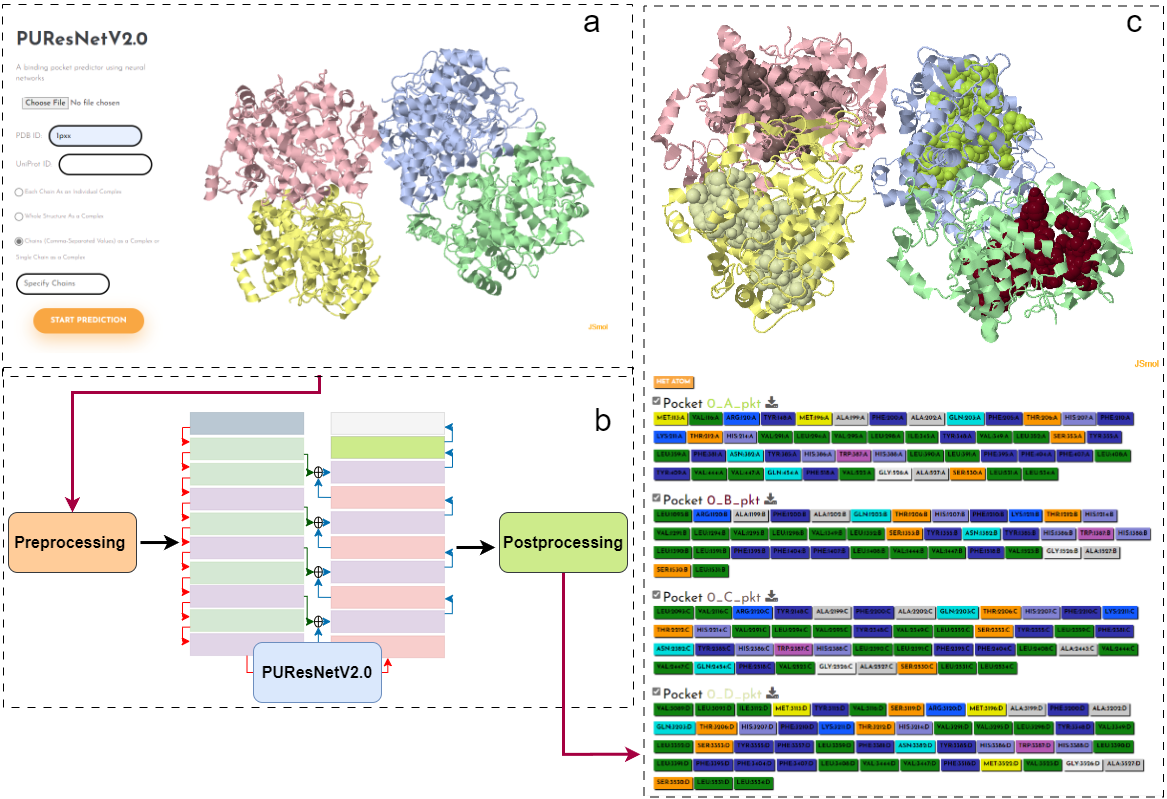
**

The webserver operates with three main steps.

1. **Select Prediction Options:** Upload a Protein Data Bank (PDB) file or enter a PDB ID or a UniProt ID. The prediction mode can be set to treat the input as a single complex, each chain as a separate complex, or specific chains (indicated by comma-separated chain identifiers) as a single complex.
2. **Backend Processing**: The webserver creates a sparse tensor from the input PDB file. It uses PUResNetV2.0 to predict binding pockets and then postprocesses these predictions for visualization purposes.
3. **Result Visualization:** The predicted protein structure and its binding pockets are displayed using JSmol ^1^. The amino acids in each pocket are identified, and the predicted pocket can be downloaded as a PDB file.

The webserver can be accessed on <http://nsclbio.jbnu.ac.kr/tools/jmol> using any browser of choice. We recommend using a chromium engine-based browser. Accessing the webserver using tablets and mobile devices is not recommended since it is not optimized for those devices.

#
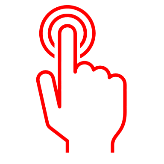
User Input Options


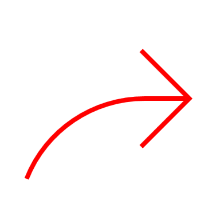

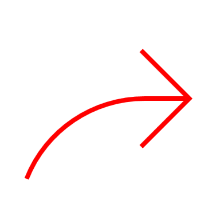

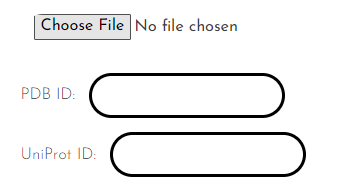


e.g., 5urv

e.g., P05067

Users can input protein structure data in three ways, located on the left side of the homepage.

1. **Upload a Protein Structure in the .pdb File Format:** Users can directly upload a protein structure in the widely accepted PDB file format. This method is most suitable for custom-designed models or structures that are not yet available in public databases.
2. **Provide a Valid PDB ID**: Inputting a valid PDB ID allows the tool to fetch the associated protein structure from the RCSB Protein Data Bank. This method is ideal for users wishing to analyze publicly available protein structures.
3. **Provide a Valid UniProt ID:** By providing a valid UniProt ID, the tool can retrieve the corresponding protein structure from the AlphaFold database ^2, 3^. This option is useful when an experimentally determined protein structure is unavailable but a predicted structure from AlphaFold is available.

# Prediction Mode


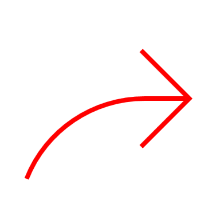

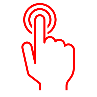

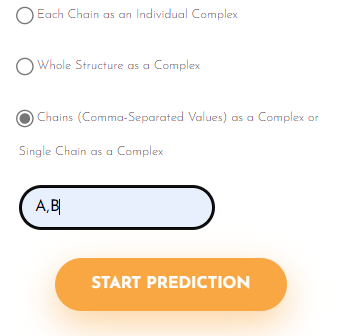


A,B,C or A,B or A (A, B and C are chain identifiers)

Three binding site prediction mode options are available.

1. **Each Chain as an Individual Complex**: In this mode, each chain in the protein structure is treated separately, and binding site prediction is performed independently for each chain.
2. **Whole Structure as a Single Complex:** The entire protein structure (i.e., all chains) is treated as a single entity for binding site prediction purposes.
3. **Chains (Comma-Separated Values) as a Complex or Single Chain as a Complex:** The chains to include in the binding site prediction process are specified by providing a comma-separated list of chain identifiers. The specified chains are treated as a single complex during the prediction process.

# Visualization and Accessing the Results


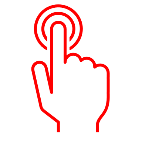

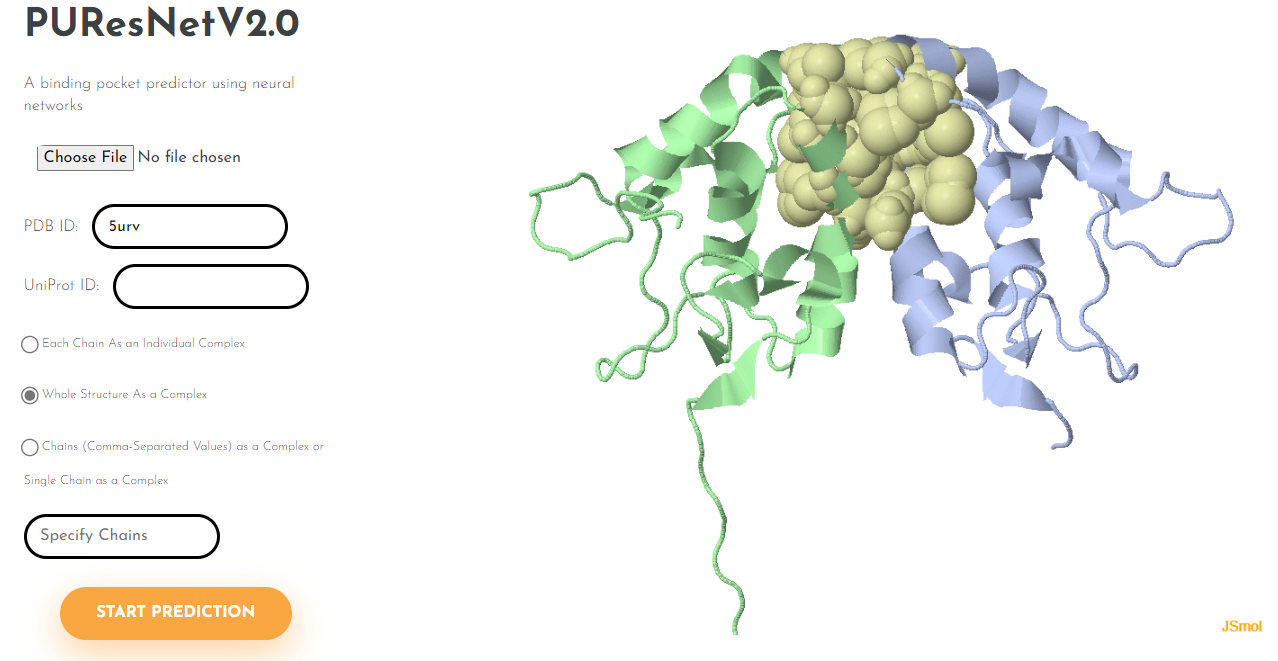


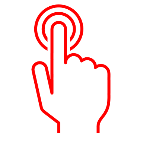
After performing the prediction process, the protein structures and the predicted binding pockets are visualized using JSmol, an open-source HTML5-based molecular viewer. The protein structures are shown as cartoons, ligands are shown as sticks, and the predicted bindings pocket are shown as spheres.


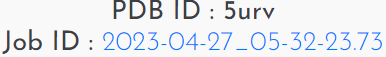


Each job performed by the user is assigned a unique identifier. This is shown after the prediction step is performed below the START PREDICTION button.


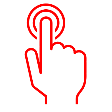

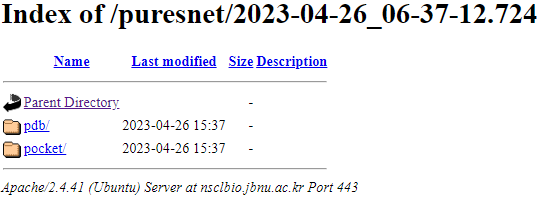


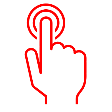

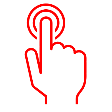

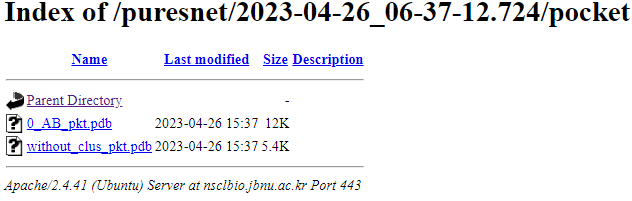


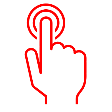
Users can access their predictions by clicking on the corresponding identifier, which opens a directory containing the individual predicted pockets and a PDB file (without_clus_pkt.pdb) with all predicted pockets (without clustering).


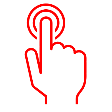

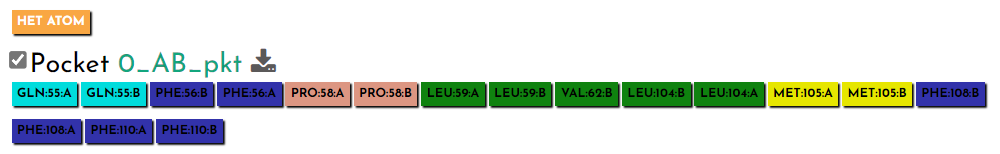


Detailed residue information is available for each predicted pocket.


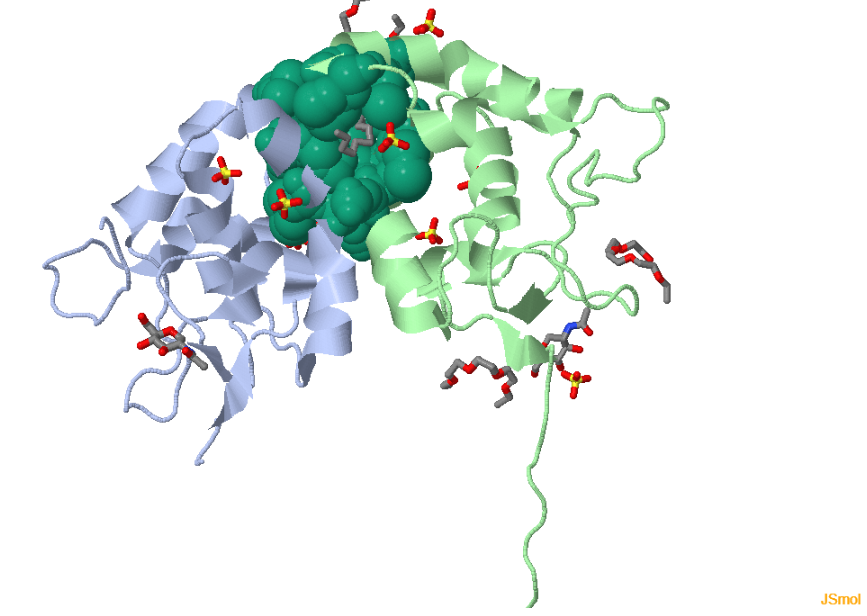


The "HET ATOM" button reveals any nonstandard atoms or molecules in the selected structure.


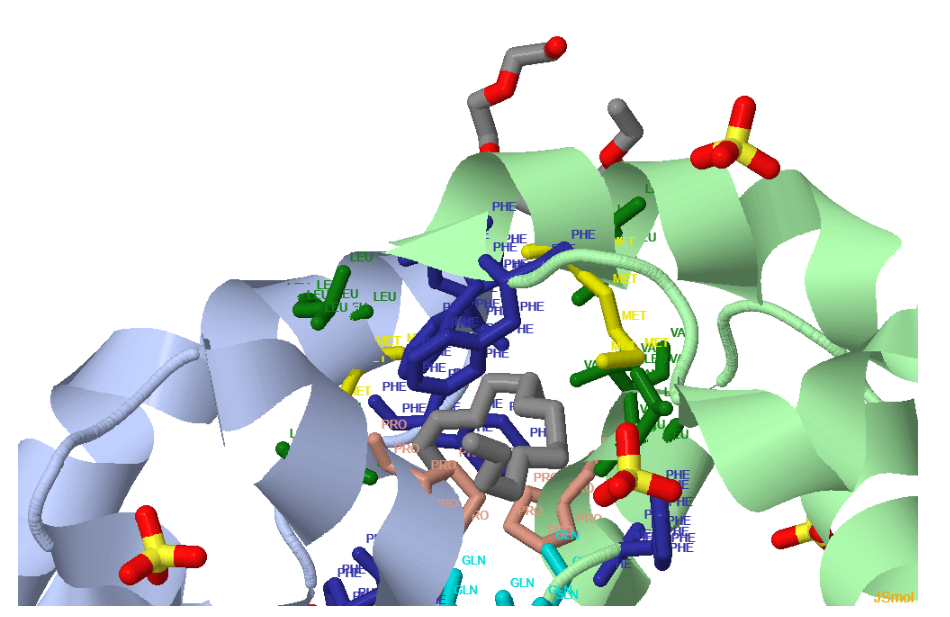


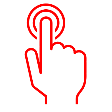
Each amino acid has a corresponding button; when clicked, the respective residues are displayed in the structure.


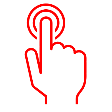

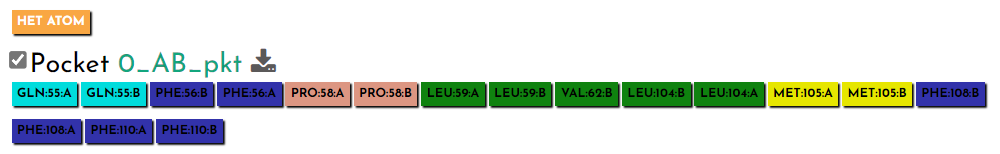


The "Pocket” checkbox enables the selective display of individual predicted pockets, which is useful for focusing on specific regions. Users can download PDB files of individual pockets by clicking on their associated “*_pkt” buttons.
